# White matter microstructure in children and adolescents with ADHD

**DOI:** 10.1016/j.nicl.2022.102957

**Published:** 2022-02-07

**Authors:** Michael Connaughton, Robert Whelan, Erik O'Hanlon, Jane McGrath

**Affiliations:** a Dept of Psychiatry, School of Medicine, Trinity College Dublin, Ireland; b School of Psychology, Trinity Dublin, Ireland; c Trinity College Institute of Neuroscience, Trinity Dublin, Ireland; d Dept of Psychiatry, School of Medicine, Royal College of Surgeons in Ireland, Dublin, Ireland

**Keywords:** Attention deficit hyperactivity disorder, Children and adolescents, Diffusion MRI, White matter microstructure, Tractography, Connectomic

## Abstract

Attention deficit hyperactivity disorder (ADHD) is a common neurodevelopmental disorder. Advances in diffusion magnetic resonance imaging (MRI) acquisition sequences and analytic techniques have led to growing body of evidence that abnormal white matter microstructure is a core pathophysiological feature of ADHD. This systematic review provides a qualitative assessment of research investigating microstructural organisation of white matter amongst children and adolescents with ADHD. This review included 46 studies in total, encompassing multiple diffusion MRI imaging techniques and analytic approaches, including whole-brain, region of interest and connectomic analyses. Whole-brain and region of interest analyses described atypical organisation of white matter microstructure in several white matter tracts: most notably in frontostriatal tracts, corpus callosum, superior longitudinal fasciculus, cingulum bundle, thalamic radiations, internal capsule and corona radiata. Connectomic analyses, including graph theory approaches, demonstrated global underconnectivity in connections between functionally specialised networks. Although some studies reported significant correlations between atypical white matter microstructure and ADHD symptoms or other behavioural measures there was no clear pattern of results. Interestingly however, many of the findings of disrupted white matter microstructure were in neural networks associated with key neuropsychological functions that are atypical in ADHD. Limitations to the extant research are outlined in this review and future studies in this area should carefully consider factors such as sample size, sex balance, head motion and medication status.

## Highlights

• A systematic review of diffusion MRI studies in children and adolescents with ADHD.
• 46 studies included, encompassing multiple diffusion MRI techniques.
• Reduced white matter microstructure was reported in several studies.
• Mixed evidence linking white matter differences with specific cognitive processes.
• Common limitations included sample size, head motion and medication status.

## 1 Introduction

Attention deficit hyperactivity disorder (ADHD) is a neurodevelopmental disorder characterised by hyperactivity, impulsivity, and inattention, which causes significant functional impairment (Thapar and Cooper, 2016). It is one of the commonest childhood psychiatric conditions with an estimated prevalence of 5.3% in children and adolescents (Polanczyk et al., 2007), and is highly heritable, with a heritability estimate of 0.76 (Faraone and Larsson, 2019). While the pathophysiology of ADHD is not well understood, neuroimaging research has reported abnormalities in both brain structure (Valera et al., 2007, Frodl and Skokauskas, 2012, Hoogman et al., 2017, Hoogman et al., 2019), function (Castellanos and Proal, 2012, Cortese et al., 2012) and functional connectivity (a term describing the co-ordination of processing or communication between brain regions (Gao et al., 2019) across widespread brain regions in children and adolescents with ADHD. The developmental periods of childhood and adolescence are of particular interest in ADHD as research has shown changes in the ADHD symptomology and neuropsychological functioning as an individual enters puberty (Dorn, 2006). Furthermore, white matter is particularly sensitive to remodelling with exposure to pubertal hormones (Juraska and Willing, 2017) and adolescence is a crucial period for the re-organisation of white matter in the brain (Paus et al., 2008).

Diffusion magnetic resonance imaging is a technique that enables the assessment of the underlying architectural organisation of white matter tracts through the measurement of restricted diffusion of water molecules in tissue (Jones and Leemans, 2011). In early 2000′s the most common diffusion MRI analysis model was Diffusion Tensor Imaging (DTI) (Basser et al., 1994, Mori and van Zijl, 2002). DTI remains an important diffusion MRI modelling technique and frequent diffusion indices using DTI modelling are fractional anisotropy; mean diffusivity, radial diffusivity and axial diffusivity (see topic box 1 in supplemental material). DTI analysis of diffusion data has a number of limitations, including its ability to model only a single fibre-tract per voxel (Pierpaoli et al., 2001, Behrens et al., 2007, Jeurissen et al., 2011). The limitations of DTI and the development of diffusion MRI acquisition parameters such as increased diffusion-weighted directions and multiple b-values have led to more advanced diffusion MRI imaging models. These include diffusion kurtosis imaging (DKI), diffusion spectrum imaging (DSI), constrained spherical deconvolution (CSD), Q-ball, fixel-based analyses (FBA) and neurite orientation and dispersion density imaging (NODDI) (Pierpaoli et al., 2001, Behrens et al., 2007, Jeurissen et al., 2011, Zhang et al., 2012, Alexander, 2008, Broad et al., 2019, Van Hecke et al., 2016). These diffusion modelling techniques estimate the fibre orientation distribution function for CSD, or diffusion orientation distribution function for DSI and Q-ball, parameters that can describe the direction of diffusion in voxels with multiple crossing fibres. Metrics derived from these higher order models have increased accuracy, yielding clinically relevant information that cannot be obtained from the DTI model (Van Hecke et al., 2016). Common metrics derived from these advanced DWI methods are summarised in topic box 1 (see supplemental table).

There are many different methods of diffusion analysis, which can be broadly categorised as follows: whole brain, region of interest, and connectomic. Whole-brain analyses evaluate local voxel-wise differences across the whole brain. A common whole-brain analysis technique is tract-based spatial statistics, an automated analysis for evaluating diffusion metrics in major white matter tracts on a voxel-wise level across groups of subjects (Smith et al., 2006). Region of interest analyses are based on the delineation of predefined areas of interest in the brain. Common region of interest techniques include atlas-based analyses and tractography. Atlas-based analysis uses a standard or population-specific atlas to evaluate differences in regions of the brain. Tractography uses the orientation of the diffusion profile to reconstruct specific white matter tracts in 3-dimensional space, allowing researchers to investigate the micro-structural organisation of white matter tracts connecting specific brain regions.

Connectomic analyses is a technique which models the human brain as a complex network (connectome) and evaluates the topological property of this network enabling the investigation of white matter organisation at the macroscopic level (Liao et al., 2017, Sporns et al., 2005). Typically, in white matter connectomic research, using both structural and diffusion MRI, the brain connectome consists of nodes comprised of nodes (grey matter) and edges (white matter) (Bullmore and Sporns, 2012). Graph Theory is a mathematical framework that can be used for the assessment and representation of the human brain connectome. A variety of graph-theoretical measures can be extrapolated that provide summary information on properties of the brain network (or sub-networks) (Bullmore and Sporns, 2012, Sporns et al., 2000) (see topic box 1 in supplemental material).

Previous *meta*-analyses of diffusion MRI research in children with ADHD reported wide-spread abnormalities in white matter microstructure. These abnormalities were in brain regions including the corpus callosum, cingulum, inferior and superior longitudinal fasciculus, inferior fronto-occipital fasciculus, uncinate fasciculus, internal capsule, cerebellum, basal ganglia and areas of the frontal, temporal, parietal and occipital lobe (van Ewijk et al., 2012, Chen et al., 2016). However, these meta-analyses only included studies that had used a whole-brain diffusion MRI approach. There has been no previous systematic review that investigated the white matter microstructure of ADHD across multiple diffusion MRI analytic techniques. This paper provides a systematic review of diffusion MRI studies that have used whole-brain, region of interest and connectomic approaches to investigate white matter microstructure in children and adolescents with ADHD. The results of this systematic review are described in the following sections: 1) whole-brain and region of interest studies, 2) connectomic studies, 3) associations between white matter and ADHD symptoms. In the discussion we explore the evidence for, and the possible impact of, disrupted white matter in the neural networks associated with the key neuropsychological functions that are atypical in ADHD.

## 2 Material and methods

A systematic literature search of the EMBASE, Medline, PsychINFO, Web of Science, and the Cochrane Library databases was conducted on the 18th of June 2021. Reference lists of retrieved studies were also searched manually to screen for additional papers. The search strategy was prospectively registered to PROSPERO, where full details and breakdown of the search strategy are available *(PROSPERO ID:* CRD42020160401*)*

**Fig. 1:**
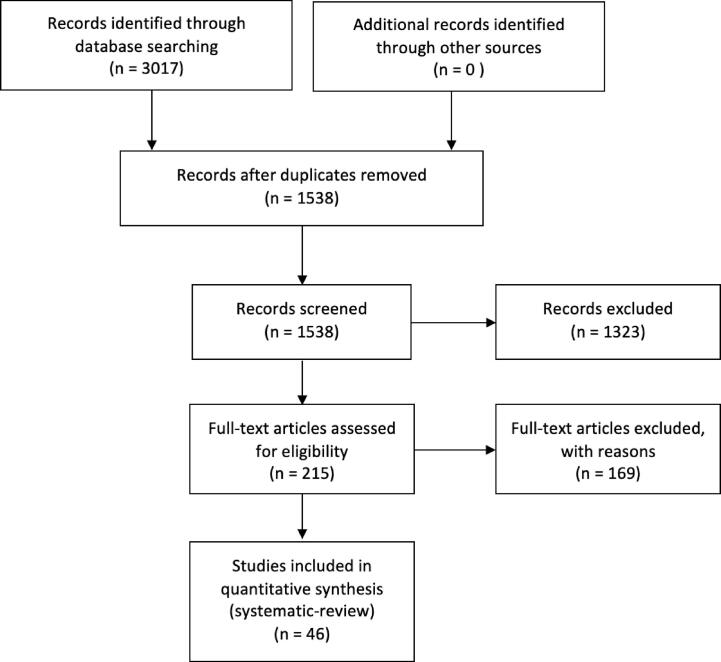
Flow diagram of selection of studies.

After de-duplication, the title and abstract of 1538 papers were screened, and relevant studies were selected and reviewed. Inclusion criteria were: human research that investigated between-group white matter differences using diffusion-weighted MRI, in children aged 3–18, who had a formal diagnosis of ADHD according to DSM-4, DSM-4-TR, DSM-5 or ICD-10. Studies were included only if they included a typically developing comparison group aged 3–18, were published in English and in a peer-reviewed journal. After screening, 1323 records were excluded, and two authors (MC and JM) independently reviewed 215 studies that met inclusion criteria to confirm eligibility. 46 studies met inclusion criteria (see Fig. 1).

The following information was extracted: study population characteristics (i.e., sample demographics, sample size, diagnostic criteria), diffusion MRI modelling technique, diffusion MRI analysis technique, main findings (Table 1). Data extraction was completed independently by the two authors MC and JM, and disagreements regarding extracted data or study inclusion were resolved by a mediator (EOH/RW) (see Fig. 1). A qualitative review of all eligible studies was then conducted.

## 3 Results

### 3.1 Whole-brain and region of interest studies

#### 3.1.1 Frontostriatal White matter tracts

There were nine studies that examined frontostriatal tracts. Seven studies reported reduced white matter microstructure in all four frontostriatal tracts (striatum-dorsolateral prefrontal cortex, striatum-orbitofrontal cortex, striatum-medial prefrontal cortex, and striatum-ventrolateral prefrontal cortex) (see Topic box 2 in supplemental material) in children and adolescents with ADHD, characterised by reduced generalised fractional anisotropy (Chiang et al., 2015, Chiang et al., 2016, Shang et al., 2013, Gau et al., 2015, Wu et al., 2014, Lin et al., 2014, Tung et al., 2021). Two studies did not find between-group differences in white matter organisation of these tracts (de Zeeuw et al., 2012, Silk et al., 2016).

#### 3.1.2 Corpus callosum

Twelve studies investigated the corpus callosum in ADHD. Eight studies reported decreased organisation of white matter microstructure in regions of the corpus callosum in young people with ADHD which was characterised by reduced fractional anisotropy (Cao et al., 2010, Qiu et al., 2011, Ameis et al., 2016, King et al., 2015, Pastura et al., 2016, Wu et al., 2017) and higher mean kurtosis (Adisetiyo et al., 2014). In contrast, one study reported increased axial diffusivity (Tamm et al., 2012). Four other studies did not report a difference in white matter organisation in this tract in ADHD (Hamilton et al., 2008, Peterson et al., 2011, Bouziane et al., 2018, Fuelscher et al., 2021).

#### 3.1.3 Superior longitudinal fasciculus

Of the sixteen studies that examined the superior longitudinal fasciculus, twelve reported reduced white matter microstructural organisation in the superior longitudinal fasciculus in children and adolescents with ADHD. These studies included reports of decreased generalised fractional anisotropy (Chiang et al., 2015, Chiang et al., 2016), decreased fractional anisotropy (Pastura et al., 2016, Wu et al., 2017), increased mean diffusivity (Pavuluri et al., 2009, Nagel et al., 2011, Lawrence et al., 2013), increased radial diffusivity (Wu et al., 2017), increased mean kurtosis (Adisetiyo et al., 2014) and decreased return-to-orientation probability and return-to-axis probability (Wu et al., 2020). In contrast, two other studies reported higher fractional anisotropy (Silk et al., 2009) and lower mean diffusivity (Adisetiyo et al., 2014) in the superior longitudinal fasciculus amongst individuals with ADHD. Two studies did not find between-group differences in white matter organisation of these tracts (Peterson et al., 2011, Bouziane et al., 2018).

#### 3.1.4 Cingulum Bundle

Fourteen studies investigated white matter microstructure of the cingulum. Six studies described reduced white matter organisation in the cingulum in children with ADHD characterised by reduced generalised fractional anisotropy (Chiang et al., 2015, Chiang et al., 2016, Tung et al., 2021), fractional anisotropy (King et al., 2015) and increased mean diffusivity (Pavuluri et al., 2009), and decreased return-to-orientation probability and return-to-axis probability (Wu et al., 2020). Two other studies reported conflicting results; one reported an increase in fractional anisotropy (Silk et al., 2009), the other reported higher axial diffusivity (Svatkova et al., 2016) in the cingulum in children and adolescents with ADHD. Six other studies that isolated the cingulum bundle did not report any significant between-group difference in white matter structure (Lin et al., 2014, Hamilton et al., 2008, Peterson et al., 2011, Fuelscher et al., 2021, Lawrence et al., 2013, Cooper et al., 2015).

#### 3.1.5 Thalamic white matter

Ten studies examined thalamic white matter; four of these reported a reduction in microstructural organisation of the thalamic radiation in participants with ADHD characterised by lower generalised fractional anisotropy (Tung et al., 2021), lower fractional anisotropy (Bouziane et al., 2018), higher mean kurtosis (Adisetiyo et al., 2014) and higher mean diffusivity and axial diffusivity (Lawrence et al., 2013). In contrast four studies found increased fractional anisotropy in the anterior (Tamm et al., 2012, Svatkova et al., 2016) and posterior thalamic radiation (Pastura et al., 2016, Peterson et al., 2011) in children with ADHD. Two other studies reported atypical white matter microstructure in white matter tracts connecting the thalamus to a number of regions. Reduced fractional anisotropy was found in white matter tracts between the thalamus and striatum, hippocampus, motor cortex and prefrontal cortex (Xia et al., 2012). Decreased return-to-orientation probability and return-to-axis probability was reported in white matter connections between the thalamus and pre-central gyrus, superior frontal gyrus and left paracentral gyrus (Wu et al., 2020). Increased return-to-orientation probability and return-to-axis probability was found between the thalamus and right paracentral gyrus (Wu et al., 2020).

#### 3.1.6 Internal capsule

Of the eight studies examining the internal capsule, seven found disrupted organisation of white matter in children with ADHD. Five reported decreased fractional anisotropy in the internal capsule (Qiu et al., 2011, Pastura et al., 2016, Wu et al., 2017, Ashtari et al., 2005) and posterior limb of the internal capsule (Nagel et al., 2011). Two other studies reported increased mean kurtosis, reflecting increased complexity in tissue microstructure (Adisetiyo et al., 2014), reduced fibre coherence and increased mean diffusivity (Pavuluri et al., 2009) in this tract. One study failed to find between-group differences in white matter organisation in the internal capsule (Peterson et al., 2011).

#### 3.1.7 Corona radiata

Eight studies investigated the corona radiata in children and adolescents with ADHD. Five of these reported disrupted organisation of corona radiata white matter. Reduced fractional anisotropy was reported in all regions of the corona radiata (anterior, superior and posterior) (Qiu et al., 2011, Wu et al., 2017, Pavuluri et al., 2009, Nagel et al., 2011), and increased radial diffusivity (Wu et al., 2017), axial diffusivity (Tamm et al., 2012), and mean diffusivity was reported in the anterior corona radiata (Pavuluri et al., 2009). Three other studies reported contrasting findings with increased fractional anisotropy in the anterior corona radiata (Tamm et al., 2012, Davenport et al., 2010) and reduced mean diffusivity in the superior and posterior corona radiata in children with ADHD (Adisetiyo et al., 2014).

#### 3.1.8 White matter organisation in other regions

Other white matter tracts have not been as extensively studied, and for many tracts there has been mixed findings relating to white matter microstructure with some studies finding between-group differences but others failing to find a difference. Reduced organisation of white matter microstructure has been reported in the arcuate fasciculus (Chiang et al., 2016, Tung et al., 2021), inferior longitudinal fasciculus (Pavuluri et al., 2009); uncinate fasciculus (Tung et al., 2021, Tamm et al., 2012, Fuelscher et al., 2021, Nagel et al., 2011), inferior fronto-occipital fasciculus (Tung et al., 2021, Pastura et al., 2016, Adisetiyo et al., 2014, Tamm et al., 2012, Fuelscher et al., 2021); corticospinal tract (Hamilton et al., 2008, Fuelscher et al., 2021), external capsule (Pastura et al., 2016, Wu et al., 2017, Adisetiyo et al., 2014); fronto-pontine tract (Fuelscher et al., 2021); parieto-occipital pontine tract (Fuelscher et al., 2021), frontal aslant tract (Tung et al., 2021), perpendicular fasciculus (Tung et al., 2021); stria terminalis (Tung et al., 2021); forceps major (Lin et al., 2020) and forceps minor (Qiu et al., 2011, King et al., 2015, Lawrence et al., 2013, Svatkova et al., 2016) as well as in white matter tracts in the parahippocampal gyrus (Peterson et al., 2011), lingual gyrus (Peterson et al., 2011); striatum (Wu et al., 2017, Ashtari et al., 2005); premotor region (Ashtari et al., 2005), motor cortex (Jacobson et al., 2015), basal ganglia (Qiu et al., 2011, Li, 2010), fornix (Davenport et al., 2010); fronto-parietal tracts (Nagel et al., 2011) and white matter in the medial orbitofrontal cortex (Jacobson et al., 2015), parieto-occipital region (Ashtari et al., 2005); cerebellar peduncle (Ashtari et al., 2005, Bechtel et al., 2009) and cerebellum (Nagel et al., 2011). Increased white matter microstructural organisation has been reported in the corticospinal tract (Silk et al., 2009); uncinate fasciculus (Tamm et al., 2012, Silk et al., 2009), inferior fronto-occipital fasciculus (Tamm et al., 2012), inferior longitudinal fasciculus (Silk et al., 2009, Svatkova et al., 2016), corticospinal tract (Svatkova et al., 2016), striatum (Peterson et al., 2011), anterior forceps (Tamm et al., 2012) and forceps minor (Tamm et al., 2012, Lawrence et al., 2013), as well as in white matter in the frontal region (Davenport et al., 2010, Li, 2010), and temporo-occipital white matter (Kobel, 2010). A number of studies reported no-between group difference in the inferior longitudinal fasciculus (Hamilton et al., 2008); uncinate fasciculus (Hamilton et al., 2008, Lawrence et al., 2013), inferior fronto-occipital fasciculus (Hamilton et al., 2008, Lawrence et al., 2013); corticospinal tract (Cooper et al., 2015), cerebellar peduncle (Fuelscher et al., 2021), forceps major (Lawrence et al., 2013) and the basal ganglia (Silk et al., 2009).

### 3.2 Connectomic studies

Five studies were identified that used graph theory analysis to investigate both global and regional white matter microstructure in children with ADHD. In graph theory analysis of whole brain networks, children with ADHD displayed the same small-world network organisation seen in a neurotypical population (Cao et al., 2013, Beare et al., 2017), but decreased global, long-range connections suggesting a reduction in connections between local, functionally specialised networks in ADHD (Cao et al., 2013, Beare et al., 2017, Cha et al., 2015). The greatest reduction in efficiency was seen in the left parietal, frontal, and occipital cortices (Cha et al., 2015). Decreased white matter organisation was reported inside highly connected regions (rich-club regions) of the network amongst children with ADHD (Ray et al., 2014). These results suggest that ADHD may be characterised by under-connectivity inside highly connected regions (rich-club regions) and that this underconnectivity may be partially explained by findings of lower generalised fractional anisotropy within these regions (Ray et al., 2014). However, the white matter networks in the population with ADHD were not simply characterized by reduced connectivity; outside of highly connected regions (rich-club regions), white matter microstructure between other brain regions was increased, highlighting the complexity of the network dynamics within this disorder (Ray et al., 2014). Regional abnormalities of the connectome in children with ADHD were characterised by reduced connectivity in a network comprising frontal, striatal, and cerebellar regions (Hong et al., 2014), decreased white matter connections in prefrontal circuitry (Cao et al., 2013, Beare et al., 2017) and fronto-accumbal circuitry (Cha et al., 2015), and increased white matter connections in the orbitofrontal-striatal circuitry in children with ADHD (Cao et al., 2013).

### 3.3 Associations between white matter and ADHD symptoms

Several of the diffusion MRI studies included in this systematic review investigated the relationship between white matter organisation and ADHD symptom severity.

#### 3.3.1 Overall ADHD severity

Greater severity of ADHD symptoms has been correlated with increased fractional anisotropy in the cingulum bundle (Cooper et al., 2015) and left sagittal stratum (Peterson et al., 2011), and with greater left lateralisation of fractional anisotropy values in white matter between the putamen and ventrolateral prefrontal cortex (Silk et al., 2016). Overall severity has been associated with reduced fibre density in the left fronto-pontine tract (Fuelscher et al., 2021) and reduced axonal/cellular density and volume in the thalamus-precentral gyrus bundle (Wu et al., 2020). However, other studies have failed to find any correlation between diffusion metrics and ADHD severity scores (Bouziane et al., 2018, Svatkova et al., 2016, Ercan et al., 2016).

#### 3.3.2 Inattention

Higher inattention scores were significantly associated with reduced generalised fractional anisotropy in the left striatum-orbitofrontal cortex (Wu et al., 2014), right superior longitudinal fasciculus (Chiang et al., 2015) and cerebellum (Ashtari et al., 2005), increased mean diffusivity, axial diffusivity, or radial diffusivity values in the forceps minor (Lawrence et al., 2013) and reduced network connectivity strength in a prefrontal network (Cao et al., 2013). A number of other studies have reported correlations between inattention scores and white matter organisation in the frontostriatal tracts (Chiang et al., 2016, Shang et al., 2013), superior longitudinal tracts (Chiang et al., 2016), cingulum bundle (Chiang et al., 2015, Chiang et al., 2016), posterior corona radiata, posterior limb of the internal capsule, frontolimbic and temporo-occipital white matter (Nagel et al., 2011) however, these studies have not specified the direction of the association.

#### 3.3.3 Hyperactivity/impulsivity

Higher scores on measures of hyperactivity/impulsivity have been associated with increased fractional anisotropy in the right inferior longitudinal fasciculus (King et al., 2015), corpus callosum, right superior longitudinal fasciculus and right corona radiata (Wu et al., 2017), lower fractional anisotropy in the forceps major (Lin et al., 2020) and increased network connectivity strength in the orbitofrontal-striatal portion of a defined network (Cao et al., 2013). Other studies reported significant associations between hyperactivity/impulsivity and white matter organisation in frontostriatal tracts (Chiang et al., 2015, Shang et al., 2013, Wu et al., 2014), superior longitudinal fasciculus (Chiang et al., 2015) and cingulum bundle (Chiang et al., 2015) although the direction of association has not been specified. One study did not find any correlation between white matter microstructure and hyperactivity/impulsivity scores in ADHD (Hamilton et al., 2008).

#### 3.3.4 Other neuropsychological/behavioural functions

Reduced organisation of white matter microstructure in young people with ADHD has also been significantly associated with deficits in a variety of neuropsychological functions including executive function (Chiang et al., 2016, Lawrence et al., 2013, Svatkova et al., 2016), vigilance (Chiang et al., 2015, Wu et al., 2014), cognitive control (Li, 2010), inhibitory control (Jacobson et al., 2015), fine motor competence (Hyde et al., 2021), delayed reward (Bessette and Stevens, 2019) and school dysfunction (Gau et al., 2015). Significant correlations have been reported between white matter microstructural organisation and spatial planning (Chiang et al., 2016, Shang et al., 2013); reaction time (Lin et al., 2014, Fall et al., 2015) and short-term memory (Chiang et al., 2016), but the direction of these correlations has not been specified.

## 4 Discussion

### 4.1 Overall findings

The results of this systematic review highlight widespread abnormalities of white matter microstructure in both discrete white matter tracts and neural networks in children and adolescents with ADHD. Whole-brain and region of interest approaches reported atypical organisation of white matter microstructure in several white matter tracts, with the most prominent findings in the frontostriatal tracts, corpus callosum, superior longitudinal fasciculus, cingulum bundle, thalamic radiations, internal capsule and corona radiata. Connectomic approaches suggested global underconnectivity in connections between functionally specialised networks as well as regional reductions in network efficiency in frontal, parietal, striatal, occipital, and cerebellar regions. In some white matter tracts however, increased connectivity was reported and it appears that ADHD is not simply characterised by underconnectivity within neural networks, highlighting the complexity of this neurodevelopmental disorder.

From a behavioural perspective, many studies have reported significant correlations between disrupted white matter organisation and a variety of behavioural measures. However, few studies have investigated the association between the same behavioural measure and diffusion metric in the same white matter tract, and replication is therefore required. In many studies it was not clear whether correlation analyses were exploratory or whether there was correction for multiple comparisons. In addition, a number of studies did not report the direction of the association rendering the information less clinically meaningful. Consequently, there is not yet a clear consensus on the overall impact white matter pathology has on core features of ADHD or other behavioural measures.

Research in ADHD has consistently reported deficits across a wide range of neurocognitive domains. Several neuropsychological theories postulate that the core deficits of ADHD are underpinned primarily by executive dysfunction​​ (Barkley, 1997), atypical reward processing (Sagvolden et al., 2005, Tripp and Wickens, 2008, Sonuga-Barke, 2011), aberrant functioning of the default mode network (Sonuga-Barke and Castellanos, 2007), or delay aversion (Sonuga-Barke et al., 2008). While it is clear that there is atypical white matter microstructure in ADHD, the links between white matter pathology and these neuropsychological theories has not been well explored. In the following sections we provide a brief overview of four key neuropsychological theories of ADHD, consider their associated neural networks and explore potential links with the white matter pathology described in this review. There is overlap in certain white matter tracts which are involved in multiple neurocognitive processes (e.g. frontostriatal tract, superior longitudinal fasciculus and cingulum bundle). We synthesise the findings of this systematic review to explore if neuroimaging evidence is concordant with disrupted white matter in these networks.

#### 4.1.1 Executive dysfunction theory of ADHD

The executive dysfunction theory of ADHD holds that deficits in executive function underpin the core symptoms of ADHD (Barkley, 1997). There is a wealth of behavioural and neuroimaging data supporting this hypothesis (Roth and Saykin, 2004, Hosenbocus and Chahal, 2012, Hart et al., 2013), however this theory does not provide a unifying pathophysiological explanation for ADHD (Solanto, 2001, Nigg et al., 2005, Sonuga-Barke et al., 2010, de Zeeuw et al., 2012, Sjöwall et al., 2013, Coghill et al., 2014). Executive functioning is subserved by the cortico-striato-thalamo-cortical (CSTC) neural network, superior longitudinal fasciculus white matter and the cingulum bundle. The frontostriatal tracts are a key component of the CSTC and connect the striatum to the frontal cortex. In children and adolescents with ADHD, previous research has reported reduced white matter microstructural organisation of frontostriatal tracts (Chiang et al., 2015, Chiang et al., 2016, Shang et al., 2013, Gau et al., 2015, Wu et al., 2014, Lin et al., 2014, Tung et al., 2021) was associated with inattention (Chiang et al., 2016, Shang et al., 2013); deficits in focused attention (Chiang et al., 2015), impulsivity (Chiang et al., 2015, Wu et al., 2014), school dysfunction (Gau et al., 2015), reaction time (Lin et al., 2014), hyperactivity/impulsivity (Shang et al., 2013), executive function (Shang et al., 2013) and ADHD symptom severity (Beare et al., 2017). The superior longitudinal fasciculus, and specifically the superior longitudinal fasciculus II has also been implicated in executive functioning in ADHD. The superior longitudinal fasciculus II is thought to play a role in visuospatial awareness and attention (Chiang et al., 2015, Schmahmann et al., 2008). White matter microstructure of the superior longitudinal fasciculus II was reduced (Tung et al., 2021, Wu et al., 2020) in children with ADHD and this finding has been associated with reduced fine motor control (Hyde et al., 2021). The cingulate gyrus is associated with executive function (Schermuly et al., 2010, Bubb et al., 2018) and this brain region is strongly connected to the cingulum bundle (Nolte et al., 2016). Reduced microstructural organisation of white matter in the cingulum bundle has been repeatedly reported in the ADHD literature (Chiang et al., 2015, Chiang et al., 2016, Tung et al., 2021, King et al., 2015, Nagel et al., 2011, Wu et al., 2020) and this atypical white matter has been associated with inattention (Chiang et al., 2015, Chiang et al., 2016), sustained attention (Chiang et al., 2015), impulsivity (Chiang et al., 2015), vigilance (Chiang et al., 2015), planning (Chiang et al., 2016), ADHD severity (Cooper et al., 2015), reaction time (Lin et al., 2014), executive function (Svatkova et al., 2016).

#### 4.1.2 Atypical reward processing theory of ADHD

Altered sensitivity to reward is considered a core element in the pathophysiology of ADHD (Sagvolden et al., 2005, Tripp and Wickens, 2008, Sonuga-Barke, 2011). Behaviourally, children with ADHD tend to favour small immediate rewards over larger delayed ones (Sonuga-Barke, 2011). Functional MRI studies have consistently shown that individuals with ADHD show neural hyposensitivity in dopaminergic neurons in the nucleus accumbens when presented with rewarding stimuli (Baroni and Castellanos, 2015, Plichta and Scheres, 2014, Furukawa et al., 2020).The main neural network subserving reward processing is the fronto-accumbal circuitry (Knutson et al., 2007, Cha et al., 2016), which originates in the nucleus accumbens and projects to prefrontal regions (orbitofrontal cortex, anterior cingulate gyrus, dorsal prefrontal cortex). Reduced white matter microstructure of the fronto-accumbal circuitry has been reported in youths with ADHD (Cha et al., 2015) and this white matter change has been associated with increased aggression (Cha et al., 2015). The frontostriatal tract (striatum-orbitofrontal cortex) is also a key component of the reward processing circuitry (Haber, 2011) and in children and adolescents with ADHD, a number of studies have reported reduced white matter microstructure of this tract (Chiang et al., 2015, Chiang et al., 2016, Shang et al., 2013, Gau et al., 2015, Wu et al., 2014, Lin et al., 2014) This atypical white matter has been associated with inattention (Chiang et al., 2016, Shang et al., 2013, Wu et al., 2014), focused attention (Chiang et al., 2015), hyperactivity/impulsivity (Chiang et al., 2015, Cao et al., 2013), school dysfunction (Gau et al., 2015), reaction time (Lin et al., 2014), executive function (Shang et al., 2013).

#### 4.1.3 Default mode network theory of ADHD

The default mode network (DMN) theory of ADHD suggests that many problems associated with ADHD arise from periodic lapses in attention due to spontaneous intrusions of DMN activation (Sonuga-Barke and Castellanos, 2007). The DMN is a network comprised of distinct brain regions in the ventromedial and lateral prefrontal cortex, posteromedial and inferior parietal cortex, and medial and lateral temporal cortex (Andrews-Hanna et al., 2010, Kernbach et al., 2018, Lopez-Persem et al., 2019). The nodes of the DMN are connected by a number of major white matter tracts including the anterior and posterior cingulum bundles, uncinate fasciculus, superior longitudinal fasciculus II, arcuate fasciculus, and inferior longitudinal fasciculus (Alves et al., 2019). There are also structural connections between subcortical and cortical nodes of the DMN; fibres of the anterior thalamic radiation connect the thalamus and prefrontal cortex, fibres of the cingulum connect the basal forebrain with the prefrontal and cingulate cortices and fibres of the fornix connect the basal forebrain with the hippocampus (Alves et al., 2019). Reduced organisation of white matter microstructure has been described in the cingulum (Chiang et al., 2015, Chiang et al., 2016, Tung et al., 2021, King et al., 2015, Pavuluri et al., 2009, Nagel et al., 2011, Wu et al., 2020); superior longitudinal fasciculus II (Tung et al., 2021, Wu et al., 2020); arcuate fasciculus (Chiang et al., 2016, Tung et al., 2021) and inferior longitudinal fasciculus (Nagel et al., 2011). Conversely, an increase in white matter microstructural organisation was reported in the uncinate fasciculus (Tamm et al., 2012) and anterior thalamic radiate (Tamm et al., 2012, Lawrence et al., 2013, Svatkova et al., 2016). White matter microstructural organisation in the DMN tracts was also significantly associated with ADHD symptoms and neuropsychological functioning; in the cingulum (inattention (Chiang et al., 2015, Chiang et al., 2016), sustained attention (Chiang et al., 2015), impulsivity (Chiang et al., 2015), vigilance (Chiang et al., 2015), planning (Chiang et al., 2016), ADHD severity (Cooper et al., 2015), reaction time (Lin et al., 2014) and executive function (Svatkova et al., 2016)), superior longitudinal fasciculus II (motor response (Cortese et al., 2012)), arcuate fasciculus (inattention (Chiang et al., 2016) and executive functioning (Chiang et al., 2016)) and inferior longitudinal fasciculus (delay reward (Bessette and Stevens, 2019), impulsivity (King et al., 2015), executive functioning (Svatkova et al., 2016)).

#### 4.1.4 Delay aversion theory of ADHD

The delay aversion theory holds that a desire to avoid delay underpins the core deficits in ADHD (Sonuga-Barke et al., 2008, Sonuga-Barke, 2005), delay aversion is mediated by atypical functioning in brain regions associated with the anticipation and response to aversive outcomes. These regions are primarily the amygdala and its connections with the prefrontal cortex (specifically dorsolateral prefrontal cortex and ventrolateral prefrontal cortex), temporal pole, and insula (Sonuga-Barke, 2005, Van Dessel et al., 2018). Functional MRI research findings have provided support for the delay aversion theory (Van Dessel et al., 2018, Lemiere et al., 2012, Wilbertz et al., 2013, Van Dessel et al., 2020). Research specifically investigating the white matter microstructure of the complete delay aversion network in ADHD has not yet been conducted. However significant abnormalities have been found in components of this network. The uncinate fasciculus is a major white matter tract connecting the amygdala and the ventral prefrontal cortex; greater white matter microstructure of the uncinate fasciculus predicted reduced amygdalar activation (Kim and Whalen, 2009, Swartz et al., 2014). Three studies included in this systematic review reported atypical white matter organisation in the uncinate fasciculus. However the findings were mixed with two study finding reduced generalised fractional anisotropy (Tung et al., 2021) and fractional anisotropy (Nagel et al., 2011) and the other finding increased fractional anisotropy (Silk et al., 2009). Impairment in an individual’s ability to wait for future rewards has been associated with reduced activation in brain reward circuitry, specifically in the ventral striatum and dorsolateral prefrontal cortex (Bessette and Stevens, 2019, Van Dessel et al., 2018, Bishop, 2008, Gold et al., 2015). Much previous research has described a reduction in white matter microstructural organisation of the frontostriatal-dorsolateral tract in children with ADHD (Chiang et al., 2015, Chiang et al., 2016, Shang et al., 2013, Gau et al., 2015, Wu et al., 2014, Lin et al., 2014) , a finding that has been associated with focused attention (Chiang et al., 2015), sustained attention (Chiang et al., 2015), hyperactivity (Shang et al., 2013), vigilance (Chiang et al., 2015), reaction time (Lin et al., 2014), school dysfunction (Gau et al., 2015).

In summary, this systematic review is the first review paper to synthesise evidence of atypical white matter microstructure in children and adolescents with ADHD in relation to the neuropsychological theories of ADHD (executive functioning, reward processing, delay aversion and default mode network functioning). Disrupted organisation of white matter may be a neurobiological feature that could potentially provide a unifying pathophysiological account for the diverse neuropsychological theories of ADHD.

### 4.2 Limitations of diffusion MRI research in ADHD

Key limitations include variance in sample demographics, sample size, head motion and medication status.

#### 4.2.1 Sample demographics

In relation to study populations, it is important to consider sex and age range and a recent study using normative modelling to investigate white matter in ADHD and autism spectrum disorder suggested that some of the inconsistencies in findings might be explained by confounders of age and sex (Tung et al., 2021). There was a significant sex imbalance in many studies included in this systematic review, with 12/45 studies including only males in their sample. In the developing brain the effects of sex on white matter microstructure remain unclear but may influence diffusion MRI measures (Bava et al., 2011, Chiang et al., 2011). The heterogenous age ranges found in the studies included in this review may limit the ability to compare results across different studies. White matter organisation is sensitive to remodelling in childhood and particularly in adolescence (Juraska and Willing, 2017) and white matter in younger children with ADHD may have changed significantly by later adolescence.

#### 4.2.2 Sample size

Small sample sizes, typical for brain-wide association studies (research linking differences in brain structure to behavioural phenotypes), may be a key element in the widespread replication failure of brain-wide association studies (Button et al., 2013, Ioannidis et al., 2014, Botvinik-Nezer et al., 2020). It is hoped that the increased sample size facilitated by datasets from large consortia will increase the reproducibility of brain-wide association studies.

#### 4.2.3 Head motion

Many diffusion MRI studies in children with ADHD have not controlled for head motion which may lead to false positive findings (Aoki et al., 2018). Head motion has been associated with a spurious reduction in FA (Yendiki et al., 2014), a finding that raises concern that results of some diffusion MRI research may be a result of group differences in head motion. This would be particularly pertinent for a condition such as ADHD where hyperactivity is a core feature. It is important that future ADHD neuroimaging research considers the impact of head motion, controlling for head motion both during scanning and during image processing.

#### 4.2.4 ADHD medication

Another factor that may contribute to the differences in findings between studies is the impact of ADHD medication on brain structure. It has been suggested that unmedicated children with ADHD display reduced white matter volume compared to both neurotypical controls and medicated children with ADHD (Castellanos, 2002). A recent clinical trial found that following four months of methylphenidate treatment, boys with ADHD had an increase in FA in several association tracts and the corpus callosum compared to non-medicated boys with ADHD (Bouziane et al., 2019). A study included in this systematic review investigated drug-naïve boys with ADHD finding no case-control differences in white matter microstructure (Bouziane et al., 2018), with the authors suggesting that previously seen case-control differences may partially be attributed to medication use. As studies typically contain participants with mixed medication status it is important that future research considers the potential effects of medication.

## 5 Conclusion

This paper was a systematic review of diffusion MRI research in children and adolescents with ADHD. Our results showed that white matter microstructural organisation was disrupted in many major fibre tracts in young people with ADHD, however there is heterogeneity in the literature that may stem from a variety of methodological limitations. There is not yet a clear consensus about the impact of white matter pathology on core features of ADHD or other behavioural measures, but this review has shown that numerous studies have reported aberrant white matter in the neural networks associated with four key neuropsychological theories of ADHD. Atypical white matter microstructure appears to be a core neurobiological feature of ADHD which could provide a unifying pathophysiological explanation for major neuropsychological theories of ADHD.

## Declaration of Competing Interest

The authors declare that they have no known competing financial interests or personal relationships that could have appeared to influence the work reported in this paper.
