# Supplementary material for: White matter microstructure in children and adolescents with ADHD
Source: Neuroimage Clin. 2022 Feb 7;33:102957. doi: 10.1016/j.nicl.2022.102957 (PMC8842077; doi:10.1016/j.nicl.2022.102957)
Supplement: Supplementary data 1 [file mmc1.docx]

Topic box 1: Diffusion MRI metrics

| **Modelling technique** | **Metric** | **Description** |
| --- | --- | --- |
| ***DTI*** | *Fractional anisotropy* | A summary measure that characterises the degree of anisotropy in each voxel. Reduced fractional anisotropy may reflect demyelination, axonal damage or loss of white matter coherence^1,2^. |
|  | *Radial diffusivity* | A measure of diffusivity perpendicular to the principle direction of diffusion. Increased radial diffusivity is believed to be a sensitive marker for demyelination^1,3^. |
|  | *Axial diffusivity* | A measure of the diffusion along the principal axis of the diffusion. Reduced axial diffusivity is thought to reflect disrupted axonal integrity^1,4^. |
|  | *Mean diffusivity* | A measure of the total amount of diffusion in a voxel, regardless of direction. Increased mean diffusivity is thought to reflect oedema, axonal loss, and demyelination^1,4^. |
| ***DSI*** | *Generalised fractional*  *Anisotropy* | An analogue to fractional anisotropy, a summary measure that characterises the degree of anisotropy in each voxel^1^. |
|  | *Return-to-orientation probability* | Measures total diffusional cellular volume^5-7^. |
|  | *Return-to-axis probability* | Describes axonal density and packing^5-7^. |
| ***DKI*** | *Mean kurtosis* | An analogue to mean diffusivity, a measure of total diffusion kurtosis regardless of direction. Reflecting microstructural complexity^1^. |
|  | *Axial Kurtosis* | The diffusion kurtosis along the axial direction^8^. |
|  | *Radial Kurtosis* | The diffusion kurtosis along the radial direction^8^. |
| ***FBA*** | *Fibre density* | A measure of white matter microstructure^9,10^. |
|  | *Fibre cross-section* | A fixel-wise analogue of tensor-based morphometry^9^. |
|  | *Fibre density and cross-section* | A fixel-wise analogue of voxel-based morphometry^9^. |
| ***NODDI*** | *Neurite density index* | A measure of neurite density ^11-13^. |
|  | *Orientation dispersion index* | A measure of fanning of neurites ^11-13^. |
|  | *Isotropic volume fraction* | A measure of the extracellular component of the free-water compartment ^11-13^. |
| **Connectomics** | *Streamline count* | A measure of structural connectivity that quantifies the number of connections between two nodes^14^. |
| ***Graph theory*** | *Clustering coefficient* | A measure of how much neighbours of a given node are interconnected^15^. |
|  | *Path length and efficiency* | A measure of the potential for information transmission, determined as the average shortest path length across all pairs of nodes^15^. |
|  | *Centrality* | Measured by the number of connections of a node (degree centrality) or the combined weight of these connections (centrality strength) provide information of the potential of individuals nodes and edges to influence the global network^16^. |

Topic box 2: Anatomy of key white matter tracts

| **Tract name** | **Description** |
| --- | --- |
| ***Frontostriatal***  ***white matter*** | The frontostriatal tracts connect regions of the frontal lobe to the striatum in the basal ganglia and is subdivided into four tracts: striatum-dorsolateral prefrontal cortex, striatum-orbitofrontal cortex, striatum-medial prefrontal cortex, and striatum-ventrolateral prefrontal cortex ^17^. |
| ***Corpus callosum*** | The largest commissural fibre that connects both hemispheres of the brain. It is subdivided into four components (genu, body, rostrum, isthmus and splenium) ^18^. |
| ***Superior longitudinal fasciculus*** | The superior longitudinal fasciculus is the largest association fibre tract and connects the frontal lobe to the parietal, temporal, and occipital lobes. It can be divided into three components superior longitudinal fasciculus I, II and III^19^. |
| ***Cingulum bundle*** | The cingulum bundle connects frontal, parietal, and medial temporal sites, and also links the subcortical nuclei to the cingulate gyrus ^18^. |
| ***Thalamic white matter*** | The thalamus is the primary relay centre of the brain^20^. The major white-matter fibre projections from the thalamus are the thalamocortical radiations, which connect the thalamus to the cerebral cortex ^21^. |
| ***Internal Capsule*** | The internal capsule connects the cerebral cortex and subcortical structures, brainstem, and spinal cord ^22^. |
| ***Corona radiata*** | White matter from the internal capsule projects to the cerebral cortex via the corona radiata white matter, which is divided into anterior, superior and posterior sections ^18^. |

Supplementary Table: Summary of study characteristics and results

| **Author** | **ADHD Group:**  **n, mean age (range or SD)** | **Control Group**  **n, mean age (range or SD)** | **% Male** | **Diffusion MRI Modelling Technique** | **Diffusion MRI Analysis Technique** | **Regions investigated** | **Between-group white matter differences** | **Behavioural Correlations in ADHD** |
| --- | --- | --- | --- | --- | --- | --- | --- | --- |
| Bechtel et al., 2009 | N=14  10.43 (1.34) | N=12  10.92(1.62) | 100% | DTI | ROI (atlas- based) | • middle cerebellar | ↓ middle cerebellar peduncle (right) | N/A |
| Bouziane et al., 2018 | N=60  11.34(0.87) | N=60  11.36(0.84) | 100% | DTI | ROI (atlas-based  TBSS | **ROI (atlas-based)**  •corpus callosum  •anterior thalamic radiation  •superior longitudinal fasciculus  **TBSS**  •Whole brain analysis | No significant between group differences | No significant correlation was found between symptom severity and diffusion metrics |
| Cao et al., 2010 | N=28  13.3(1.5) | N=27  13.2(0.9) | 100% | DTI | ROI (atlas based) | • Corpus callosum  -genu  -rostral  -anterior  -posterior  -isthmus  -splenium | ↓isthmus | N/A |
| Fall et al., 2015 | N=11  (8-13) | N=11  (8-13) | 82% | DTI | ROI (atlas based) | • caudate  • putamen  • pallidum  • thalamus | No significant between group differences | Mean reaction time positively correlated with MD in caudate (bilateral), putamen (right) and thalamus (right)  Intra-individual standard deviation of reaction time was positively correlated with MD in b-caudate, b-putamen, b-thalamus |
| Hamilton et al., 2008 | N=17  11.96 (2.32) | N=16  11.72 (2.48) | 100% | DTI | ROI (atlas- based) | **ROI (atlas-based)**  •cingulum bundle  •corpus callosum  •corticospinal tract  •fornix  •optic radiations  •uncinate fasciculus  •superior longitudinal fasciculus  •superior occipitalfrontal fasciculus  •inferior occipitalfrontal fasciculus | ↓ corticospinal tract  ↓ superior longitudinal fasciculus (bilateral) | No significant correlation was found between hyperactivity and diffusion metrics |
| Peterson et al., 2011 | N=16  11.28(1.55) | N=16  11.15(2.14) | 69% | DTI | ROI (atlas-based)  Voxel-based analysis (whole brain) | **ROI (atlas-based)**  •cingulum bindle  •corpus callosum (body, splenium, genu)  •anterior thalamic radiation  •posterior thalamic radiation  •superior longitudinal fasciculus  •anterior limb of internal capsule  •posterior limb of internal capsule  •superior fronto-occipital fasciculus  •sagittal striatum  **Voxel-based analysis**  •whole brain analysis | **ROI (atlas-based)**  ↑ striatum (left)  **Voxel-based analysis** ↑superior frontal gyrus (right)  ↑posterior thalamic radiations (right)  ↑dorsal posterior cingulate gyrus (left)  ↑lingual gyrus (left)  ↑parahippocampal gyrus (left) | Total ADHD symptom severity positively associated with FA in the sagittal striatum (left) |
| Silk et al, 2009 | N=15  12.6(2.4) | N=15  12.6(2.4) | 100% | DTI | ROI (manual) | •basal ganglia (caudate, putamen/globus pallidum)  •thalamus | No significant between group differences | No significant correlation was found between symptom severity and diffusion metrics |
| Cha et al., 2015 | N=19  10.1(2.0) | N=31  10.5(2.1) | 70% | DTI | Tractography | •fronto-accumbal network  -NAcc-medial orbitofrontal cortex  - NAcc-lateral orbitofrontal cortex  - NAcc-medial PFC  - NAcc-frontal pole  - NAcc-rostral ACC  - NAcc-pars orbitalis | ↓NAcc-lateral OFC (left) | NAcc-medial PFC (left) was positively associated with aggression  NAcc-rACC (left) was negatively associated with aggression |
| Chiang et al., 2015 | N=50  11.26(2.93) | N=50  11.22(2.79) | 76% | DSI | Tractography | •fronto-striatal tracts  -caudate-VLPFC  -caudate-DLPFC  -caudate-OFC  •superior longitudinal fasciculus  •cingulum bundle | ↓caudate-VLPFC (left)  ↓caudate-DLPFC (left)  ↓caudate ate-OFC (left)  ↓superior longitudinal fasciculus (bilateral)  ↓cingulum bundle (right) | Inattention symptoms were negatively correlated with GFA in the caudate-VLPFC (right), caudate-DLPFC (bilateral), SLF (bilateral) and cingulum bundle (bilateral).  Focused attention was significantly associated with GFA in the caudate-DLPFC (left) and caudate-OFC (right).  Impulsivity was significantly associated with GFA in the caudate-OFC (left), SLF (right) and cingulum bundle (left).  Sustained attention was significantly associated with GFA in the caudate-DLPFC (bilateral), SLF (right), cingulum bundle (right).  Vigilance was significantly associated with GFA in the caudate-DLPFC (left) and cingulum bundle (left). |
| Chiang et al., 2016 | N=45  11.36(2.86) | N=45  11.29(2.71) | 73% | DSI | Tractography | •fronto-striatal tracts  -striatum-VLPFC  -striatum-DLPFC  -striatum-OFC  •superior longitudinal fasciculus  •cingulum bundle  •arcuate fasciculus | ↓striatum-VLPFC (left)  ↓striatum-DLPFC (left)  ↓striatum-OFC (left)  ↓superior longitudinal fasciculus (left)  ↓arcuate fasciculus (left)  ↓cingulum bundle (right) | Inattention symptoms were negatively correlated with GFA in the striatum-VLPFC (bilateral), striatum-DLPFC (right), striatum-OFC (right), superior longitudinal fasciculus (bilateral), arcuate fasciculus (bilateral) and cingulum bundle (bilateral).  Executive functioning was significantly associated with GFA in the superior longitudinal fasciculus and arcuate fasciculus.  Planning was significantly associated with GFA in the cingulum bundle. |
| Cooper, Thapar & Jones et al., 2015 | N=17  15.6(1.3) | N=17  16.9(1.2) | 100% | CSD | Tractography | •subgenual cingulum  •corticospinal tract | No significant between group differences | ADHD severity was positively correlated with FA in the subgenual cingulum (left).  ADHD severity was negatively correlated with RD in the subgenual cingulum (left).  Autistic trait was negatively correlated with RD in the inferior segment of the corticospinal tract (right). |
| DeZeeuw et al., 2012 | N=30  9.6(2.3) | N=34  10.2(2.3) | 89% | DTI | Tractography | •striatum-prefrontal cortex | No significant between group differences | No significant correlation was found between attention and diffusion metrics |
| Fuelscher et al., 2021) | N=76  (9.4-11.5) | N=68  (9.6-11.9) | 65% | HARDI | Tractography | •anterior commissure  •corpus callosum  •cerebellar projections  •cingulum bundle  •cortico-spinal tract  •front-pontine tract  • inferior fronto-occipital fasciculus  •inferior longitudinal fasciculus  •middle longitudinal fasciculus  •parieto-occipital pontine tract  •superior longitudinal fasciculus  •striatal projectios  •thalamic projections  •uncinate fasciculus | ↓ cortico-spinal tract (bilateral)  ↓ front-pontine tract (bilateral)  ↓ inferior fronto-occipital fasciculus (left)  ↓ uncinate fasciculus (right)  ↓ parieto-occipital pontine tract (right) | Greater ADHD symptom severity was associated with lower FD in the front-pontine tract (left) |
| Gau et al., 2015 | N=32  11.4(2.3) | N=32 | 91% | DSI | Tractography | • fronto-striatal tracts  -caudate-VLPFC  -caudate-DLPFC  -caudate-OFC  -caudate-MPFC | ↓caudate-VLPFC (bilateral)  ↓caudate-DLPFC (bilateral)  ↓ caudate -OFC (bilateral)  ↓ caudate -OFC (bilateral) | School dysfunction was significantly correlated with GFA in the caudate-OFC (bilateral), caudate-DLPFC (right) and caudate-VLPFC (right). |
| Hyde et al., 2021 | N=55  9.60(0.42) | N=61  9.58(0.45) | 64% | HARDI | Tractography | •SLF I  •SLF II  •SLF III | N/A | Slower motor response in non-dominant hand was significantly associated with reduced apparent fibre density in the SLF I (right) and reduced volume in the SLF I, II, III (right) |
| Lawrence et al., 2013 | N=56  12.6(3.2) | N=17  13.2(2.0) | 62% | DTI | Tractography | •anterior thalamic radiations  •cingulum bundle  •corticospinal tract  •inferior fronto-occipital fasciculus  •inferior longitudinal fasciculus  •forceps major  •forceps minor  •superior longitudinal fasciculus  •uncinate fasciculus | ↓anterior thalamic radiation  ↓forceps minor  ↓superior longitudinal fasciculus | MD, AD and RD of the forceps minor were correlated with intention scores. |
| Lin et al., 2014 | N=28  11.54(2.30) | N=28  11.57(2.75) | 89% | DSI | Tractography | •fronto-striatal tracts  -caudate-VLPFC  -caudate-DLPFC  -caudate-OFC  -caudate-MPFC  •cingulum bundle | ↓caudate-VLPFC (bilateral)  ↓caudate-DLPFC (bilateral)  ↓ caudate -OFC (bilateral)  ↓ caudate -OFC (bilateral) | GFA in the caudate-MPFC (left), caudate-OFC (left, caudate-VLPFC (left) and cingulum bundle (bilateral) was significantly associated with reaction time. |
| Shang et al., 2013 | N=25 | N=25 | Not given | DSI | Tractography | •fronto-striatal tracts  -caudate-VLPFC  -caudate-DLPFC  -caudate-OFC  -caudate-MPFC | ↓caudate-VLPFC (bilateral)  ↓caudate-DLPFC (bilateral)  ↓ caudate -OFC (bilateral)  ↓ caudate -OFC (bilateral) | Inattention was significantly associated with GFA in caudate-OFC (right)  Hyperactivity/impulsivity was significantly associated with GFA in caudate-DLPFC (left) and caudate-MPFC (right)  Executive functions were significantly associated with caudate-OFC (left) and caudate-VLPFC (left) |
| Silk et al., 2016 | N=21  13.3(1.8) | N=22  14.6(2.2) | 100% | HARDI | Tractography | • fronto-striatal tracts  -caudate-VLPFC  -caudate-DLPFC  -caudate-OFC  -putamen-VLPFC  -putamen -DLPFC  -putamen -OFC | No significant between group differences in specific white matter tract  hemisphere lateralization  ↓caudate-VLPFC (right)  ↓caudate-DLPFC (right)  ↓putamen-VLPFC (left) | N/A |
| Tung et al., 2021 | N=147  11.2(2.4) | N=Not given  < 16.0 | Not given | DSI | Tractography | •arcuate fasciculus  •superior longitudinal fasciculus I,II,III  •frontal aslant tracts  •perpendicular fasciculus  •cingulum, body  •cingulum, hippocampus  •fornix  •stria terminalis  •uncinate fasciculus  •inferior fronto-occipital fasciculus  •frontostriatal circuit (prefrontal)  •frontostriatal circuit (motor)  •thalamocortical radiations (prefrontal cortex)  •thalamocortical radiations (sensorimotor)  •thalamocortical radiations (auditory)  •thalamocortical radiations (optic)  •corticospinal tract  •corpus callosum (prefrontal)  • corpus callosum (sensorimotor)  • corpus callosum (parietal)  • corpus callosum (temporal)  • corpus callosum (occipital) | ↓arcuate fasciculus (right)  ↓superior longitudinal fasciculus II (right)  ↓superior longitudinal fasciculus III (right)  ↓frontal aslant tracts (right)  ↓perpendicular fasciculus (left)  ↓cingulum, body (left)  ↓cingulum, hippocampus (left)  ↓stria terminalis (left)  ↓uncinate fasciculus (left)  ↓inferior fronto-occipital fasciculus (bilateral)  ↓frontostriatal circuit (prefrontal) (bilateral)  ↓frontostriatal circuit (motor) (bilateral)  ↓thalamocortical radiations (prefrontal cortex) (left)  ↓thalamocortical radiations (sensorimotor) (left)  ↓thalamocortical radiations (auditory) (bilateral) | N/A |
| Wu et al., 2014 | N=25  11.36(2.14) | N=25  11.40(2.69) | 88% | DSI | Tractography | •fronto-striatal tracts  -caudate-VLPFC  -caudate-DLPFC  -caudate-OFC  -caudate-MPFC | ↓caudate-VLPFC (bilateral)  ↓caudate-DLPFC (bilateral)  ↓ caudate -OFC (bilateral)  ↓ caudate -OFC (bilateral) | GFA of the caudate-OFC (left) was negatively correlated with inattention  GFA of caudate-VLPFC (left) was significantly associate with impulsivity. |
| Wu et al., 2020 | N=30  10.64(1.69) | N=28  10.61(1.73) | 71% | HARDI | Tractography | •cortico-striatal-thalamic fibres (45 in total)  •SLF II  •cingulum bundle | ↓SLF-II (right)  ↓thalamus-precentral gyrus (right)  ↓thalamus-superior frontal gyrus (right)  ↓caudate-medial orbitofrontal gyrus (right)  ↓ caudate-precentral gyrus (right)  ↓thalamus-paracentral-gyrus (left)  ↓caudate-caudal middlefrontal gyrus (left)  ↓cingulum bundle (bilateral) | Increased symptom severity was significantly associated with lower axonal/cellular density and volume in the thalamus-precentral (right) |
| Xia et al., 2012 | N=19  10.9(2.3) | N=19  12.2(2.3) | 63% | DTI | Tractography | • thalamus-striatum  • thalamus-hippocampus  • thalamus-amygdala  • thalamus-prefrontal cortex  • thalamus-motor cortex  • thalamus-somatosensory cortex  • thalamus-posterior parietal cortex  • thalamus-temporal cortex  • thalamus-occipital cortex | ↓thalamus-striatum (bilateral)  ↓thalamus-motor cortex (left)  ↓thalamus-hippocampus (left)  ↓thalamus-prefrontal cortex (right) | N/A |
| Ashtari et al., 2005 | N=18  8.94(1.5) | N=15  9.13(1.4) | 64% | DTI | Voxel-based analysis (whole brain) | Whole-brain analysis | ↓premotor (right)  ↓striatum (right)  ↓cerebellar peduncle (right)  ↓middle cerebellar peduncle (left)  ↓parieto-occipital regions (left) | N/A |
| Davenport et al., 2010 | N=14  15.0(2.34) | N=26  14.8(2.41) | 70% | DTI | Voxel-based analysis (whole brain) | Whole-brain analysis | ↓posterior fornix (left)  ↑inferior prefrontal regions (left)  ↑superior prefrontal regions (right) | N/A |
| Jacobson et al., 2015 | N=60  10.2(1.1) | N=60  9.9(1.3) | 50% | DTI | Voxel-based analysis (whole brain) | Whole-brain analysis | ↓M1 (bilateral)  ↓mOFC cortex (bilateral) | Decreased FA in M1 was associated with reaction time  Lower FA in mOFC was associated with reaction time. |
| Kobel et al., 2010 | N=14  10.43(1.34) | N=12  10.92(1.62) | 100% | DTI | Voxel-based analysis (whole brain) | Whole-brain analysis | ↑temporo-occipital white matter (left) | N/A |
| Li et al., 2010 | N=20  10.12(1.83) | N=24  9.62(2.19) | 91% | DTI | Voxel-based analysis (whole brain) | Whole-brain analysis | ↑frontal white matter (right) | Interference control was positively correlated with FA in frontal white matter (right)  Verbal fluency was negatively correlated with frontal white matter (right) |
| Qiu et al., 2011 | N=15  12.65(1.82) | N=15  13.21(1.73) | 100% | DTI | Voxel-based analysis (whole brain) | Whole-brain analysis | ↓forceps minor (bilateral)  ↓internal capsule (bilateral)  ↓corona radiata (bilateral)  ↓corpus callosum (splenium)  ↓basal ganglia (bilateral) | N/A |
| Adisetiyo et al., 2014 | N=27  12.9(2.8) | N=27  13.3(2.6) | 50% | DKI | Voxel-based analysis (whole brain)  TBSS | •Whole brain analysis | **Voxel-based analysis**  No significant between group differences  **TBSS**  ↓superior longitudinal fasciculus (bilateral)  ↓anterior corona radiata (bilateral)  ↓superior corona radiata (bilateral)  ↓cingulate (left)  ↓posterior thalamic radiations (left)  ↓sagittal striatum (bilateral)  ↓Inferior longitudinal fasciculus (bilateral)  ↓Inferior fronto-occipital fasciculus (bilateral)  ↓external capsule (bilateral)  ↓internal capsule (anterio, posterior and retrolenticular limb) (bilateral)  ↓corpus callosum (genu, body, splenium) (bilateral)  ↑superior longitudinal fasciculus (right)  ↑corona radiata (superior, posterior) (right) | N/A |
| Ameis et al., 2016 | N=31  10.3(1.8) | N=62  10.8(2.8) | 70% | DTI | TBSS | Whole-brain analysis | ↓corpus callosum | N/A |
| Bessette & Stevens, 2019 | N=67  15.41(1.78) | N=68  15.43(1.73) | 77% | DTI | TBSS | Whole-brain analysis | N/A | Delay aversion was negatively associated with FA in CC, ACR (bilateral), IC (bilateral), PCR (bilateral), SLF (right), ILF (left). |
| Ercan et al., 2016 | N=72  10.73(8-15) | N=24  10.8(8-15) | 81% | DTI | TBSS | Whole-brain analysis | No significant between group differences | No significant correlation was found between symptom severity and diffusion metrics |
| King et al., 2015 | N=19  12.68(2.14) | N=24  14.42(2.76) | 51% | DTI | TBSS | Whole-brain analysis | ↓superior longitudinal fasciculus (bilateral)  ↓forceps minor  ↓cingulum bundle (left)  ↓callosal regions (bilateral) | Impulsivity score was positively correlated with FA in the inferior longitudinal fasciculus (right) among females with ADHD |
| Nagel et al., 2011 | N=20  8.05(0.69) | N=16  8.31(0.70) | 45% | DTI | TBSS | Whole brain analysis | ↓fronto-limbic white matter (bilateral)  ↓tempero-occipital white matter (bilateral)  ↓cerebellar white matter (left)  ↓fronto-parietal white matter (right)  ↓anterior corona radiata (right)  ↓superior corona radiata (right)  ↓posterior corona radiata (left)  ↑superior longitudinal fasciculus (right)  ↑internal capsule (posterior) (right) | Inattention and hyperactivity symptoms were significantly associated with  FA  frontolimbic white matter (left)  anterior corona radiata (right)  superior corona radiata (right)  frontoparietal white matter (left)  MD  superior longitudinal fasciculus (right) |
| Pastura et al., 2016 | N=13  8(1.2) | N=14  9(1.3) | 74% | DTI | TBSS | Whole-brain analysis | ↓corpus callosum (splenium)  ↓SLF (right)  ↓internal capsule (retrolenticular)(bilateral)  ↓inferior-fronto occip[ital fasciculus (bilateral)  ↓external capsule (left)  ↓posterior thalamic radiation (left) | N/A |
| Pavuluri et al., 2009 | N=13  13.4(3.0) | N=15  13.2(2.7) | 84% | DTI | TBSS | Whole-brain analysis | ↓anterior corona radiata  ↓internal capsule (anterior, superior, posterior)  ↓superior longitudinal fasciculus  ↓inferior longitudinal fasciculus  ↓cingulum bundle | N/A |
| Silk et al., 2009 | N=15  12.6(2.4) | N=15  12.9(2.6) | 100% | DTI | TBSS | Whole-brain analysis | ↑cingulum bundle (right)  ↑uncinate fasciculus (left)  ↑inferior longitudinal fasciculus (left)  ↑superior longitudinal fasciculus (right) | N/A |
| Svatkova et al., 2016 | N=33  13.87 | N=23  14.15 | 75% | DTI | TBSS | Whole-brain analysis | ↓forceps minor  ↑cingulum bundle (bilateral)  ↑anterior thalamic radiation (left)  ↑inferior longitudinal fasciculus (left) | Executive function scores were significantly associated with FA in the inferior iongitudinal fasciculus (right) and cingulum (right). |
| Tamm, Barnea-Goraly & Reiss, (2012) | N=12  15.8(1.5) | N=12  15.6(0.8) | 100% | DTI | TBSS | Whole-brain analysis | ↑anterior corona radiata  ↑uncinate fasciculus  ↑inferior fronto-occipital fasciculus  ↑anterior thalamic radiation  ↑corpus callosum (genu)  ↑anterior forceps | N/A |
| Wu et al., 2017 | N=83  11.0(1.99) | N=122  10.6(1.81) | 65% | DTI | TBSS | Whole-brain analysis | ↓corpus callosum (genu, body, splenium)  ↓internal capsule (posterior, retrolenticular, splenium) (left)  ↓anterior corona radiata (left)  ↓superior corona radiata (left)  ↓posterior corona radiata (left)  ↓anterior corona radiata (left)  ↓posterior thalamic radiation (left)  ↓striatum(left)  ↓ external capsule (left)  ↓ superior longitudinal fasciculus (left) | Reduced inhibitory control was negatively correlated with decreased FA in the anterior corona radiata (left), superior corona radiata (left), internal capsule (left)  Reduced inhibitory control was positively correlated with increased FA posterior corona radiata (right) |
| Yoncheva et al., 2016 | N=82  10.63(2.8) | N=80  11.04(2.6) | 73% | DTI | TBSS | Whole-brain analysis | No significant between group differences | N/A |
| Beare et al., 2017 | N=21  13.28(1.86) | N=21  14.79(2.12) | 100% | CSD | Connectomic: graph theory | Whole-brain analysis | Global findings  ↓global communication  ↓long-range communication  Subnetwork finding  ↑inferior frontal regions (bilateral)  ↑middle frontal regions (bilateral)  ↑orbitofrontal regions (bilateral)  ↑precentral regions (bilateral)  ↑cingulate (bilateral)  ↑putamen (bilateral) | Symptom severity was positively associated with FA of the subnetwork encompassing,  Frontostriatal tracts (bilateral)  Occipital regions (left)  Temporal regions (left)  Parietal regions (left) |
| Cao et al., 2013 | N=13  10.3(1.9) | N=30  10.6(1.6) | 100% | DTI | Connectomic:  Graph theory | Whole-brain analysis | Global findings  ↓global efficiency  ↑shortest path length  Subnetwork finding  ↓prefrontal-dominant circuitry  ↑orbitofrontal-striatal circuitry | Decreased prefrontal-dominant circuitry was negatively correlated with inattention score.  Increased orbitofrontal-striatal circuitry was positively correlated with hyperactivity/impulsivity score. |
| Hong et al., 2014 | N=71  9.39(2.59) | N=26  10.04(2.47) | 65% | DTI | Connectomic:  Graph theory | Whole-brain analysis | Global findings  ↓connectome (consisting of ventral, frontal, basal ganglia regions) | Attention scores were negatively correlated with FA in the defined connectomes (consisting of ventral, frontal, basal ganglia regions) |
| Ray et al., 2014 | N=20  8-12 | N=20  8-12 | Not given | HARDI | Connectomic:  Rich club | Whole-brain analysis | Global findings  ↓under-connectivity inside the rich club  ↓GFA inside the rich club  ↑increased connectivity outside the rich club | N/A |

Note: DTI = diffusion tensor imaging, DSI = diffusion spectrum imaging, CSD = constrained spherical deconvolution, HARDI = high angular resolution diffusion imaging, ↓ = reduced white matter organisation, ↑ = increased white matter organisation, FA = Fractional anisotropy, MD = mean diffusivity, RD = radial diffusivity, AD = axial diffusivity, GFA = generalised fractional anisotropy, FD = fibre density, NAcc = nucleus accumbens, ACC = anterior cingulate cortex, VLPFC = ventrolateral prefrontal cortex, DLPFC = dorsolateral prefrontal cortex, OFC = orbitofrontal cortex, MPFC = medial prefrontal cortex.

References

1. Van Hecke, W., Emsell, L. & Sunaert, S. *Diffusion tensor imaging: a practical handbook*, (Springer, 2016).

2. Zhang, Y. & Burock, M.A. Diffusion tensor imaging in Parkinson's disease and parkinsonian syndrome: a systematic review. *Frontiers in neurology* **11**, 1091 (2020).

3. Song, S.K.*, et al.* Demyelination increases radial diffusivity in corpus callosum of mouse brain. *Neuroimage* **26**, 132-140 (2005).

4. Alexander, A.L., Lee, J.E., Lazar, M. & Field, A.S. Diffusion tensor imaging of the brain. *Neurotherapeutics* **4**, 316-329 (2007).

5. Assaf, Y., Mayk, A. & Cohen, Y. Displacement imaging of spinal cord using q‐space diffusion‐weighted MRI. *Magnetic Resonance in Medicine: An Official Journal of the International Society for Magnetic Resonance in Medicine* **44**, 713-722 (2000).

6. Özarslan, E.*, et al.* Mean apparent propagator (MAP) MRI: a novel diffusion imaging method for mapping tissue microstructure. *NeuroImage* **78**, 16-32 (2013).

7. Ning, L., Westin, C.-F. & Rathi, Y. Estimating diffusion propagator and its moments using directional radial basis functions. *IEEE transactions on medical imaging* **34**, 2058-2078 (2015).

8. Steven, A.J., Zhuo, J. & Melhem, E.R. Diffusion Kurtosis Imaging: An Emerging Technique for Evaluating the Microstructural Environment of the Brain. *American Journal of Roentgenology* **202**, W26-W33 (2013).

9. Raffelt, D.*, et al.* Apparent Fibre Density: a novel measure for the analysis of diffusion-weighted magnetic resonance images. *Neuroimage* **59**, 3976-3994 (2012).

10. Genc, S.*, et al.* Impact of b-value on estimates of apparent fibre density. *Human Brain Mapping* **41**, 2583-2595 (2020).

11. Zhang, H., Schneider, T., Wheeler-Kingshott, C.A. & Alexander, D.C. NODDI: practical in vivo neurite orientation dispersion and density imaging of the human brain. *Neuroimage* **61**, 1000-1016 (2012).

12. Alexander, D.C. A general framework for experiment design in diffusion MRI and its application in measuring direct tissue-microstructure features. *Magn Reson Med* **60**, 439-448 (2008).

13. Broad, R.J.*, et al.* Neurite orientation and dispersion density imaging (NODDI) detects cortical and corticospinal tract degeneration in ALS. *J Neurol Neurosurg Psychiatry* **90**, 404-411 (2019).

14. Yeh, C.-H., Smith, R.E., Liang, X., Calamante, F. & Connelly, A. Correction for diffusion MRI fibre tracking biases: The consequences for structural connectomic metrics. *NeuroImage* **142**, 150-162 (2016).

15. Bullmore, E. & Sporns, O. Complex brain networks: graph theoretical analysis of structural and functional systems. *Nature Reviews Neuroscience* **10**, 186-198 (2009).

16. Sporns, O. Graph theory methods: applications in brain networks. *Dialogues Clin Neurosci* **20**, 111-121 (2018).

17. Tekin, S. & Cummings, J.L. Frontal–subcortical neuronal circuits and clinical neuropsychiatry: an update. *Journal of psychosomatic research* **53**, 647-654 (2002).

18. Nolte, J., Vanderah, T. & Gould, D. Nolte’s the Human Brain: An Introduction to Its Functional Anatomy. (Philadelphia, PA: Elsevier, 2016).

19. Barbeau, E.B., Descoteaux, M. & Petrides, M. Dissociating the white matter tracts connecting the temporo-parietal cortical region with frontal cortex using diffusion tractography. *Sci Rep* **10**, 8186 (2020).

20. Schmahmann, J.D. Vascular syndromes of the thalamus. *Stroke* **34**, 2264-2278 (2003).

21. George, K. & Das, J.M. Neuroanatomy, thalamocortical radiations. *StatPearls [Internet]* (2021).

22. Chowdhury, F., Haque, M., Sarkar, M., Ara, S. & Islam, M. White fiber dissection of brain; the internal capsule: a cadaveric study. *Turk Neurosurg* **20**, 314-322 (2010).
